# Insulin-Like Growth Factors in the Pathogenesis of Neurological Diseases in Children

**DOI:** 10.3390/ijms18102056

**Published:** 2017-09-26

**Authors:** Raili Riikonen

**Affiliations:** Child Neurology, Children’s Hospital, University of Eastern Finland and Kuopio University Hospital, P.O. Box 1627, FI-70211 Kuopio, Finland; raili.riikonen@kolumbus.fi; Tel.: +358-50-5174696; Fax: +358-19-668418

**Keywords:** insulin-like growth factor, pathogenesis, neonates, neurodevelopmental disorders, neurodegeneration, neuroprotection, children

## Abstract

Insulin-like growth factors play a key role for neuronal growth, differentiation, the survival of neurons and synaptic formation. The action of IGF-1 is most pronounced in the developing brain. In this paper we will try to give an answer to the following questions: Why are studies in children important? What clinical studies in neonatal asphyxia, infantile spasms, progressive encephalopathy–hypsarrhythmia–optical atrophy (PEHO) syndrome, infantile ceroid lipofuscinosis (INCL), autistic spectrum disorders (ASD) and subacute sclerosing encephalopathy (SSPE) have been carried out? What are IGF-based therapeutic strategies? What are the therapeutic approaches? We conclude that there are now great hopes for the therapeutic use of IGF-1 for some neurological disorders (particularly ASD).

## 1. Introduction

The members of the insulin gene family (insulin-like growth factors 1 and 2; (IGF-1 and IGF-2, respectively) play a key trophic role in the central nervous system (CNS). Of the two, IGF-1 is more important. In fact, IGF-1 stimulates DNA synthesis, cell production, neurite outgrowth, enhances secretion of several neurotransmitters, and mediates the action of other neurotrophic factors (particularly brain derived neurotrophic factor (BDNF)).

Therefore, foetal brain growth is extremely sensitive to IGF-1 levels. Mice lacking IGF-1 or IGF-1 receptors have decreased brain volumes [1], whereas IGF-2 over-expression increases brain growth [2]. However, CNS sensitivity to IGF-1 is not the same among the different CNS regions. In fact, while cerebellar Purkinje cells and granule cells, oligodendrocytes, and motor-neurons are particularly sensitive to IGF-1 deficiency, other cells are independent of the presence of IGF-1 [3]. IGF-1 and its receptors are found widely distributed in all brain regions. In vitro studies examining the effect of IGF-1 in neural stem cells report increased neural progenitor cell proliferation and maintenance in cell cultures following treatment with IGF-1. IGF-1 increases myelination by increasing the number of myelinated axons and the thickness of myelin sheaths; moreover, IGF-1 increases both the number of oligodendrocytes and the amount of axonal myelin produced. IGF-1 increases oligodendrocyte numbers by acting as a mitogen and inhibiting apoptosis. Both neurons and glial cells have been observed to express IGF-1 mRNA, which leads to immature glial precursors developing into oligodendrocytes [4]. Oligodendrocytes mediate the myelinations of axons and constitute the vast majority of cells in the white matter. Consequently, a low production of IGF-1 may be related to the degeneration of myelin [5].

## 2. Why Studies in Children with IGF-1 Are Important?

Neurotrophic factors play a particularly important role in the developing brain. There is a dramatic growth and maturation of the brain and its different trajectories [6]. This dynamic neurodevelopment continues from the embryonic period to adolescence with significant changes in myelination, synaptogenesis and neurotransmitter distribution throughout the maturation phase. Since the development of the fetal brain requires the organized sequential expression of genes during critical periods, the fetal brain is very sensitive to insults occurring during its development. However, the developing brain also possesses a superior capacity to reorganize after insults. This is considered to be due to increased receptor sensitivity to large amounts of neurotrophic factors acting in this period of life and in early childhood [7]. Therefore, brain plasticity is greater in infants than in adults, this is the reason why it would be important to carry out therapeutic trials with neurotrophic factors such as IGF-1 in children suffering damages, commencing as soon as possible once the damage has occurred.

## 3. What Clinical Studies Have Been Carried Out?

### 3.1. Neonatal Asphyxia

Recombinant IGF-1 (rhIGF-1) is effective (neuroprotective) and non-toxic when administered 2 h after an induced hypoxic event in an animal model [8]. In fetuses as well as in older children, hypoxia reduces circulating IGF-1 [9,10]. Critical illnesses reduce circulating IGF-1 concentrations after birth. Furthermore, preterm infants have already low serum concentrations of IGF-1, and episodes of hypoxia are a frequent finding in them during the first weeks of life [11]. Preterm delivery may be precipitated by infection leading to the detection of markers of inflammation in cord blood samples, associated with reduced IGF-1 concentrations [12,13]. Acute and chronic hypoxia and inflammation also increase the concentrations of IGF-binding protein-1 (IGFBP-1). Hence the amount of free IGF-1 is lower because of its binding to IGFBP-1, and this correlates with the poor brain development observed in preterm infants [12,14]. With the development of magnetic resonance imaging (MRI), the impact of preterm birth on the different brain structures can be easily seen [15].

Moreover, postnatal head circumference correlates positively with serum IGF-1 concentrations in preterm infants [16]. Apart of these effects on the brain, IGF-1 treatment has both short-term and long-term potential benefits in many organ and systems of preterms: pulmonary and cardiovascular systems, and somatic growth [14]. Clinical trials are required to determine the risks and benefits of IGF-1 replacement in very preterm infants, and in other newborns after ischemic brain injury. An ongoing phase II trial is trying determine the safety, feasibility and potential benefits and risks of IGF-1 replacement with IGF-1/IGFBP-3 in extremely premature infants.

In our study in human newborns suffering from severe neonatal asphyxia we did not analyse cerebrospinal fluid (CSF) IGF-1, but CSF BDNF was increased and β-nerve growth factor (NGF) decreased [17,18]. Increased BDNF concentrations might counteract neuronal damage. In turn, CSF NGF concentrations might serve as a marker of asphyxia.

### 3.2. The Finnish Study: Infantile Spasms, PEHO Syndrome, INCL Autistic Spectrum Disorder (ASD): Infantile Autism and Rett Syndrome

CSF concentrations of nerve growth factors are thought to reflect concentrations in the brain more accurately than serum levels. The majority of the research studies published in this area are from our group’s work in Finland, hence they make up most of the studies reviewed. CSF IGF-1 from frozen (−70 °C) samples was measured in all these studies by radioimmunoassay with commercially available kits (Mediagnost^®^, Tubingen, Germany), according to the instructions of the manufacturer. The sensitivity of the assay was 0.02 µg/L. The CSF studies were taken as part of the diagnostic evaluation both for the patients and the age-matched controls. We included only control patients who had no conditions such as malnutrition, hepatic failure, chronic inflammation, diabetes or hypothyroidism which might have influenced the CSF concentrations of IGF-1. In addition to these controls with some minor neurological diseases we also used the results of “healthy” controls. These healthy controls were 16 age-matched children admitted to Kuopio University Hospital for surgery on the lower part of the body, to be performed under spinal anesthesia: herniotomy, urethral cystoscopy, knee arthroscopy, circumcision, and suture of traumatic wounds.

In that study we aimed to investigate the pathogenesis of some neurological disorders leading to mental retardation, both in acute and chronic diseases by examining different nerve growth factors in serum and CSF. The following neuropediatric diseases were studied: infantile spasms, INCL, PEHO, infantile autism and Rett syndrome (RTT). 

The study adhered to the tenets of the Helsinki Declaration. The Ethics Committee of the Children’s Hospital, Helsinki, and Kuopio University Hospital, Kuopio, Finland, approved the study. The study had institutional approval. Patients or their caregivers gave informed consent to the research and to publishing the results obtained.

#### 3.2.1. Infantile Spasms 

West syndrome (infantile spasms, IS) is a rare epileptic disorder with onset usually prior to the age of two years. Infantile spasms are characterized by clusters of infantile spasms with hypsarhythmia or modified hypsarrhythma on interictal electroencephalogram (EEG). It is an epileptic encephalopathy where cognitive, motor and sensory functions are altered by the epilepsy itself. The syndrome is classified into symptomatic (80%), cryptogenic (15–20%) and idiopathic (15%) groups. depending on the known etiology. The newer classification divides IS into structural-metabolic, infectious immune, genetic, and unknown groups [19].

The mechanism of infantile spasms is currently unknown, and the molecular mechanisms that lead to the long-term consequences of infantile spasms are poorly understood. Many critical questions remain, e.g., what causes mental retardation in most patients. A specific period of brain maturation is believed to be fundamental to development of IS, although there might be discrepancies between brain development and age.

It has been suggested that early stress (injury or insult) during a critical pre-, peri-, and postnatal period of high corticotrophin-releasing hormone (CRF) abundance may increase CRF synthesis and activity. [20]. CRF causes seizures during early brain development but not later. Although CRF acutely stimulates adrenocorticotrophic (ACTH) secretion, chronically elevated brain CRF desensitizes the CRF receptor and eventually decrease ACTH release [21]. 

An early brain-damaging insult (i.e., hypoxia, stroke, infection) may trigger a cascade of molecular and cellular changes. The epileptic process is considered to consist of three phases: initial insult, latency period (epileptogenesis), and recurrent seizures [22].

In symptomatic (structural) infantile spasms the spasms may have their onset several months following a pre-, peri- or immediate post-natal brain injury. This latent period presents an opportunity for therapeutic intervention.

Steroids (immunosuppressive agents) are the first-line drugs for IS. The therapeutic action of ACTH in this disorder is unknown, but it might work by downregulating the secretion of CRF and other stress hormones.

IGFs influence the entire process of neurogenesis. As described above, brain growth is extremely sensitive to levels of IGF-1. IGF-1 also reduces neuro-inflammation [23]. ACTH, glucocorticoids and a ketogenic diet can be effective therapies for infantile spasms and they all affect IGF-1 levels [24,25,26].

In our study, children with symptomatic IS had markedly low CSF IGF-1 concentrations, and significantly lower ACTH concentrations when compared with children who had an idiopathic etiology. Symptomatic IS were characterized by a history of pre, peri- or postnatal damage. Prenatal stress has in animals been shown to decrease IGF-1 [27]. In patients with infantile spasms, low CSF IGF-1 correlated with the severity and length of stress, cortical damage, poor response to therapy and poor cognition. The following hypothesis was postulated: “The brain cannot produce steroids, which stimulate secretion of IGF-1, an essential growth factor for survival of synapses high-lighting its potential use as a biomarker for disease severity. Patients with low CSF IGF-1 do not respond to therapy and there is an association between low IGF-1 levels and later worsening of mental retardation” [26] (p. 1289). An association has also been found between the decline of brain growth and poor response to ACTH [28], and, furthermore, an association between symptomatic infantile spasms and infantile autism [29] (both conditions having low CSF IGF-1, see later).

In patients with symptomatic infantile spasms, the normalisation of IGF-1 axis, directly or indirectly, may contribute to normal development. We have no information about how IGF-1 administration would affect epilepsy in children. IGF-1 activation can be beneficial or harmful depending on the stage of the disease when using the hippocampal culture model of post-traumatic epileptogenesis [30] However, in an animal model, treatment with IGF-I decreased seizure severity, increased hippocampal neurogenesis, and protected against neurodegeneration and was considered to be a possible potential treatment in temporal lobe epilepsy [31].

In summary, in infantile spasms CSF concentrations of IGF-1 at the time of presentation seems to be a biomarker of (1) treatment response and progression of epilepsy, and (2) later cognitive outcome.

#### 3.2.2. PEHO Syndrome

PEHO syndrome is a specific type of West syndrome that is characterized by progressive encephalopathy with edema, hypsarrhythmia, and optic atrophy (PEHO). Riikonen published a paper on nine families with infantile spasms in siblings [32]. The medical history of the patients in five (11 siblings) of these families were compatible with PEHO syndrome. Salonen et al. [33] described 14 patients from 11 families who had a progressive encephalopathy with early onset and called it PEHO syndrome. Somer presented diagnostic criteria for PEHO syndrome based on 14 PEHO syndrome families (19 patients) in her thesis [34]. PEHO syndrome is an early childhood, autosomal recessive developmental and epileptic encephalopathy. It is characterised by severe cerebellar atrophy as a result of an almost complete loss of granule cells. Microcephaly develops at 12 months. Somer (1993) indicated that cerebellar hypoplasia is a cardinal diagnostic feature of PEHO syndrome. Head circumference was normal at birth, but usually dropped to 2 SD below average during the first year of life. *ZNHIT3* was very recently shown to be defective in PEHO syndrome [35]. Through homozygosity mapping in Finnish families positional candidate genes were identified at the chromosome 17q17 locus in PEHO syndrome. Sanger sequencing techniques identified a homozygous missense variant, L31 > S, in the *ZNHIT3* gene (OMIM 604500) as the primary genetic cause of PEHO syndrome [35]. We studied IGF-1, nitrite, nitrate and nitric oxide, from the CSF of patients with an average age of five years. As indicated above, IGF-1 is very important for cerebellar neurons. Our results showed that the levels of IGF-1 in patients with PEHO syndrome were significantly lower than in age-matched controls [36]. The only biochemical anomalies identified so far were elevated levels of nitrate and nitrite [37] and low levels of insulin-like growth factor 1 (IGF-1) in CSF [36,38], and low high-density cholesterol in serum [39].

The other half of Somer's original patients were called PEHO-like patients. PEHO-like patients have a similar clinical disorder, but they do not have cerebellar atrophy on an MRI and lacked ophthalmologic signs [39]. They showed no reduction in CSF levels. 

#### 3.2.3. Infantile Neuronal Ceroid Lipofuscinosis (INCL)

Infantile ceroid lipofuscinosis (INCL) is a neurodegenerative disorder characterized by onset in the second year of life with rapid cognitive and motor deterioration. The EEG is isoelectric by the age of 32 months. The gene locus responsible for the disease has been mapped to chromosome 1p32 [40]. INCL is characterized by generalized cerebral atrophy, strong thalamic hypointensity in the white matter and the basal ganglia, and thin periventricular high-signal rims as shown by a T2-weighted MRI. Since diffuse cerebral gyral atrophy is so severe, the brain superficially resembles a walnut at autopsy. The brain is characterized by intralysomal accumulation of autofluorescent material mainly composed by sphingolipid activator proteins A and D, which is a secondary phenomenon [41]. 

Palmitoyl protein thioesterase (PPT) is a lysosomal enzyme that hydrolyses acyl chains from fatty “acylated” oproteins and peptides and is especially abundant in neurons [40]. Normal PPT activity is essential for the normal development of cortical neurons. INCL has been shown to result from a PPT deficiency. Many myelin proteins have been shown to be “pregnylated” and “palmitylated” [42] (p. 2660). Whether there is modulation of protein “palmitylation” by growth factors remains to be investigated.

In our study, the earliest signs of INCL were seen in all the patients including deceleration of head growth (mean age, 9.5 months), specific EEG changes and typical MRI findings [43]. 

In this neurodegenerative disease, myelination of the white matter starts and may continue in the normal way until at least five months of age, but begins to deviate before the appearance of clinical symptoms and before the age of 13 months, as revealed by quantitative assessment [43]. Loss of axons and myelin is due mainly to Wallerian degeneration [44]. Increased white matter interstitial fluid, and abnormal gliosis are thought to account for the white matter intensity seen in an MRI.

Riikonen et al. [45] suggested that insulin-like growth hormones and apoptosis might play a role in the pathogenesis of INCL. As noted above, IGF-1 levels are associated with an increased number of oligodendrocytes and myelin sheath production. IGF-1 is also anti-apoptotic and a survival factor for oligodendrocyte precursors [4]. Therefore, a decreased brain production (or supply) of IGF-1 might be responsible for the increased apoptosis. For these reasons, IGF-1 and IGFBP-3 in CSF were measured. The measurements were made at an early stage (19 months). On T2-weighted images, the difference between the cortex and white matter was diminished. Low CSF IGF-1 but normal IGFBP-3 were found in patients with INCL compared with age-matched control subjects. Also, apoptotic cell death was shown in biopsies of INCL patients using terminal deoxynucleotidyl transferase (dUTP) nick end labelling (TUNEL) staining method. Autopsy specimens were taken by brain biopsy during the 1970s for diagnostic purposes when the knowledge of INCL was poor and only a rapidly progressive encephalopathy was diagnosed. In the autopsy brain specimens, in contrast, apoptosis was hardly detectable, probably reflecting the final stage of the disease, when the cellular destruction of tissue has come to an end, and hypertrophic microglia filled the tissue.

Given that, as stated above, the IGF system is important for early brain development, myelination, and neuroprotection (preventing apoptosis), it was suggested that the pathology in INCL might be associated with low CSF IGF-1.

The low IGF-1 values seem not to be specific for INCL. In our study, we showed low CSF IGF-1 in patients with progressive cerebellar diseases, including PEHO syndrome and other forms of progressive cerebellar atrophies with severe neurological involvement [36].

In another study, we showed that IGF-1 accumulates in the brain of CLN^−/−^ knockout mice. IGF-1 accumulated in the brain with or without the complex at all studied time points. The amount crossing the blood brain barrier (BBB) was low but might, however, be sufficient to affect the physiological functions and behaviour of the subjects. Administration of protein binding-3 (PB-3) conjugated IGF-1 and IGF-1/nanoparticles gives a steadier accumulation than unbound IGF-1. Constant and sustained release may increase the bioavailability and therapeutic feasibility of IGF-1 [46].

Because of the very rapid progressive course of the disease, patients with INCL could be ideal candidates for IGF-1 therapy. The potential effects of the treatment would be seen early clinically, electro-clinically and neuro-radiologically.

#### 3.2.4. Autism

There are no treatments for the core social impairment of autistic spectrum disorder. Current treatments are directed at associated symptoms. There is now more knowledge on emerging mechanism-based therapies [47,48].

Autism is a heterogeneous condition, both in its genotype and phenotype. There are hundreds of genetic variants involved in the causation of idiopathic autism. Monogenic diseases are RTT, Fragile X syndrome, and *SHANK* 3 gene deficiency (Phelan–Mc Dermic syndrome).

A fundamental question is whether common underlying molecular pathways exist in ASD. The genes implicated in the development of autism are primarily related to synaptic and immune function [49]. “Dysregulation of activity-dependent signalling networks may have a key role in the etiology of ASD” [50] (p. 328). In autism there is aberrant control of synapse formation. It is suggested that IGF-1 increases (normalizes) IGF-1 levels which are reduced in autism at an early stage of the disease [51,52].

##### Head Growth in Autism

In autism, many studies show hippocampal, cerebellar or frontal developmental abnormalities. Disorganized cortical patches suggest a prenatal origin of autism. Dysgenetic brain development is thought to begin before 30 weeks of gestation [53], perhaps through “a signalling abnormality of some neurotrophic factor, or aberrant programmed cell death” [54] (p. 10). In post-mortem specimens of individuals with autism there is evidence of decreased numbers of cerebellar Purkinje cells. Histological studies suggest that this reduction is prenatal in origin [54,55]. Post-mortem studies also support the hypothesis that there is a combination of abnormal excess and reduction at the microstructural level ([56,57], p. 157). 

Several pathological studies have reported increased brain weight and cerebral volume in individuals with autism [58,59,60,61,62,63]. Head size was increased in about a quarter of patients.

In neonates with later ASD, head circumference at birth has been found to be normal or slightly below average, and then followed by early overgrowth during the first one or two years. However, growth abruptly slowed down thereafter and the brain volumes have been reported to be smaller than those of normal controls in their adulthood [57]. In longitudinal MRI studies with multiple scans from two years of age followed up until 4–5 years. Shen et al. demonstrated enlargement at age two years with no increase in growth rate during the subsequent two years [64]. Increased brain volume may therefore be the result of overgrowth occurring prior to the age of two years. Furthermore, according to Hazlett et al. hyper-expansion of the cortical surface area between six and 12 months of age proceed the brain volume overgrowth observed between 12 and 24 months in “high-risk” infants (infants with an older sibling with autism) who were diagnosed with autism at 24 months [65]. A deep-learning algorithm that primarily uses information on the surface areas of the brain in 6–12-month-old children predicted the diagnosis of autism in high-risk children at 24 months [65]. In this study it was demonstrated that early brain changes occur during the period in which autistic behaviours first emerge [65].

Herbert et al. suggest that the enlargement of the brain develops postnatally and is caused by a temporally modulated process. The distribution of volume changes suggests a process that alters some non-axonal component of white matter, possibly myelin. In the study by Courchesne and Pierce [57], the brain overgrowth was most prominent in the frontal and temporal lobes and in the amygdala, and more apparent in grey matter than in white matter. These patterns of enlargement are consistent with postnatal increases reported in autism. More recently, Hazlett et al. found in a longitudinal MRI study generalized cerebral cortical enlargement with disproportionate enlargement of temporal lobe white matter in the ASD group in two-year-old children [66].

It is unknown what causes abnormal head growth in autism. Early rapid postnatal overgrowth is deleterious because it coincides with the time of the development of intensive cortical synaptic connections. In autism there seems to be a disruption of normal neurobiological mechanisms leading to “premature growth without guidance” [57] (p. 157). 

##### CSF IGF-1 and IGF-2 in Autism 

The parents and other caregivers usually become aware of the aberrant development during the child’s second year of age. The diagnosis is based on clinical criteria and is usually made at age 3–4 years. Normal neurobiological processes are disturbed but it is not known what specific developmentally-important molecules might be involved in this disorder. Nelson et al. suggested that there is a relationship between abnormal growth factors (BDNF) in autism and abnormal brain growth patterns [67]. However, when the same samples were later re-evaluated with a better method, no differences existed between BDNF concentrations in children with autism and normal controls.

In our study of autistic children we measured the CSF and serum concentrations of some neurotrophic factors, such as β-neurotrophic factor (NGF), brain-derived nerve growth factor (BDNF)*,* IGF-1, and IGF-2 from serum and cerebrospinal fluid by radio-immunoassay (except for BDNF). However, the levels of BDNF in the CSF were below the limit of sensitivity of the methods used. Serum BDNF levels showed a bimodial distribution of either high or low. Of the other measures, only CSF IGF-1 differed between children with autism and normal children.

The diagnosis of the patients with autism was based on multi-disciplinary studies, and the criteria of autism were defined by International Classification of Diseases -10 (ICD-10) (World Health Organization, 1993) and Diagnostic and Statistical Manual of Mental Disorders (DMS-IV) (American Psychiatric Association, 2000) criteria. Patients were admitted to the Hospital for Children and Adolescents, Helsinki, and Department of Paediatrics, Unit of Child Neurology, Kuopio University Hospital, Kuopio, Finland. All the patients underwent extensive etiological studies. The autism of these children was considered to be idiopathic. The mean IQ of the patients was 80.

In the first study, in 11 children younger than five years we found low CSF IGF-1 concentrations compared to age-matched controls with neurological disorders. There was no difference in the IGF-2 concentrations between the two groups. None of the controls exhibited any signs of malnutrition, hepatic failure, chronic inflammation, diabetes, or hypothyroidism (conditions which might have affected the IGF-1 levels) [51]. In a larger study, we studied 26 autistic children with (median age five years and five months; range one year and 11 months to 15 years and 10 months), and 16 age-matched controls. We found that CSF IGF-1 concentrations were low in autism, but only at an early age and not in older children [52]. In this study we used “healthy” age-matched controls, children who underwent surgery with spinal anaesthesia.

Head circumference is a practical measure of brain size during the first years of life. Records are available for height, weight and head circumference from birth to the date of measurement of CSF IGF-1 in our cohort. There was no evidence of macrocephaly at the time of CSF sampling (mean age 5.5 years). Pathological brain growth is largely restricted to the first 1–3 years of life which is typically prior to the diagnosis of autism. We did not measure CSF IGF-1 at the age when the children showed acceleration of head growth from birth to 3 to 6 months of age. However, we found a positive correlation between head growth (head circumference) and CSF-1 IGF concentrations in children with autism but not in the control group [52].

##### Cerebellum and Autism

In autism, many studies show hippocampal or cerebellar developmental abnormalities. One of the most consistently abnormal brain regions has been shown to be the cerebellum. Almost all of the postmortem brains of autistic individuals studied to date have shown a significant decrease in the number of Purkinje cells [54,56,58]. Morphological and electrophysiological defects in Purkinje cells are linked to system-wide ASD-like behaviour [68]. Loss of Tsc2 in Purkinje cells was associated with autistic-like behaviour in a mouse model of tuberous sclerosis and social behaviour deficits were prevented by rapamycin treatment [69]. These findings define a molecular mechanism for a cerebellar contribution to cognitive disorders such as autism.

As described before, there is evidence that IGF-1 is essential to cerebellar development and Purkinje cell survival [70,71]. Gene expression of IGF-1 has been detected in Purkinje cells and other neurons in the developing cerebellum. Low CSF IGF-1 concentrations in children with autism may point to cerebellar abnormalities. 

Accumulating evidence indicates that the cerebellum is involved in shifting attention which in turn is important for social and cognitive development [72,73]. Disordered attention is strongly characteristic of autism at a very early age but becomes less prominent with maturation [74]. This change is in a temporal relationship with the unusual trajectory of brain growth [60,62]. In preterm infants with cerebellar damage (haemorrhage) there is a high prevalence of autism [75]. Low concentrations of IGF-1 during the critical brain spurt period might be insufficient for survival of normal Purkinje cells in the cerebellum. An early reduction of Purkinje cells may result in significantly reduced cerebellar activation. 

The cerebellum is structurally and functionally abnormal in autistic patients, as shown in many studies. 

Moreover, IGF-1 and related physiology have been implicated in disorders causing progressive cerebellar dysfunction [36,70]. 

Mills et al. have reported elevated serum levels of growth hormones (including IGF-1) in 71 children aged 4–8 years with autism and autism spectrum disorder [76]. This is not in discrepancy with our findings of low CSF IGF-1 in younger children and normal values in older children. In the study that we carried out in Finland, no correlation was detected between the serum and CSF concentrations of IGFs, thus the findings cannot be directly compared with those of the Finnish study. Furthermore, there was a difference in patient and control selection between the two studies [77]. However, both studies found a significant correlation between IGF-1 levels (in the latter study serum and in our study CSF concentrations) [52] and head circumference in autistic children and controls.

##### Serotonin and IGF in Autism

There is evidence for a key role of disturbances of the serotoninergic system in autism [78,79,80]. Serotonin has been shown to act as an early regulator of brain development. Selective serotonin uptake inhibitors (SERTIs) increased the synthesis of neurotrophic factors and improved symptoms of autism in some studies [81,82,83,84]. The brain SERT is responsible for active reuptake of serotonin molecules from the synapses, and SERT was shown to be the exact site of action of these drugs. It increases serotonin availability at synapses. To our knowledge there are no published studies on the serotonin transporter (SERT) on the brain in patients with autism. We found reduced SERT binding in the medial frontal cortex, midbrain and temporal lobes using single photon emission computed tomography (SPECT) with iodine-123 labelled *N*-(2-fluoroethyl)-2β-carbomethoxy-3β-(4-iodyphenyl)-nortropane ([^123^I] nor-β-CIT) [85]. This finding confirms the suspicion that there is disruption to serotonergic dysfunction in autism. An early decrease in serotoninergic neurons could lead to low brain concentrations of IGFs, which, once more, are critical for Purkinje cell development.

In summary, how these findings fit with the phase of brain development is unclear. Low CSF IGF-1 concentrations in children with autism at an early age may suggest a disruption of normal neurobiological mechanisms in this period. To our knowledge, our study was the first to suggest that IGF concentrations may be important in the pathogenesis of autism.

Furthermore, Anlar et al. demonstrated that IGF-1 levels in urine were significantly lower in autistic than in age-matched controls [86].

#### 3.2.5. Rett Syndrome

It has been argued that Rett syndrome (RTT) is in fact a neurodevelopmental condition rather than a neurodegenerative condition. One piece of evidence for this is that mice with induced RTT show no neuronal death. There is also lack of neurodegenerative changes in postmortem brains. RTT is a disease of synaptic dysfunction [87,88]. 

At the onset, the two conditions, RTT and autism, overlap widely. To differentiate between RTT and infantile autism in the very early stages of the disorders was not always clinically easy before the MECP2 mutation in RTT was found. However, there are a number of differences in the two syndromes, such as *head growth*: microcephaly in RTT [89], normal or macrocephaly in autism [57]; *neurons*: cholinergic neurons are affected in RTT [90], serotoninergic neurons are affected in autism [91]; *neuropathology*: frontal cortex pathology in RTT [89], cerebellum and hippocampus pathology in autism [54,55,92]; *onset*: postnatal onset in RTT [87], early prenatal origin in autism beginning before 28–30 weeks of gestational age, according to earlier reported findings examining the connections of Purkinje cells with other brain structures [53]. In RTT, the forebrain is more severely affected than other cortical areas. The basal forebrain shows reduced volume [55,93], reduced cholinergic neurons [89] reduced acetyl transferase [90], low NGF concentrations post-mortem [94], and hypometabolism of the basal forebrain [95]. In our study, patients with RTT had low levels of CSF NGF [96] which is consistent with the evidence for loss of basal forebrain cholinergic neurons in RTT. In autism, the cerebellum and hippocampus are abnormal [54,80], and the serotoninergic transmission is disturbed [78].

We have evaluated serum and CSF concentrations of NGF, BDNF, glial cell line-derived nerve growth factor (GDNF), IGF-1 and IGF-2 in RTT. However, the levels of BDNF and GDNF in the CSF were below the limit of sensitivity of the methods used.Our findings of mainly normal CSF NGF in autism, low to negligible values in RTT, decreased IGF-1 in autism and normal values in RTT, agree with the different morphological and neurochemical findings in the two syndromes [97].

### 3.3. Subacute Sclerosing Panecephalitis and Over-Expression of IGF-1 in Advanced Stage

Subacute sclerosing panencephalitis (SSPE), also known as measles encephalitis, is a rare and chronic form of progressive brain inflammation caused by a persistent infection with the measles virus. Higher IGF-1 levels in CSF of advanced SSPE (stage four) have been found, as compared to other stages of the disease [98]. Both CSF insulin and IGF-1 levels were positively correlated with serum measles immunoglobulin G (IgG). EEG abnormalities were associated with higher tau protein levels and brain autopsy studies show neurofibrillary tangles associated with tau protein. Further studies will reveal whether there are possible pathogenetic relations between IGF-1, measles IgG titers and tau protein in SSPE [98].

## 4. IGF-Based Therapeutic Strategies

In animal models, IGF-1 overexpression enhanced neurogenesis and maturation, and accelerated recovery following traumatic injury [99]. In post-traumatic injury, as in several pathologies of the CNS, inflammation of the brain, abnormal microglial function, synaptic dysfunction and decreased levels of IGF-1 coexist. Therefore, increasing IGF-1 levels in the brain might be useful for treating a number pathologies, including neurodevelopmental disorders and neurodegenerative diseases. 

Treatment with IGF-1 may similarly be applicable to autism-spectrum disorders for a wide range of symptoms such as memory, anxiety, hyperactivity, seizures and social behavior. In fact, defects in neuronal networks could be rescued by IGF-1, as shown in animal studies [100,101,102]. Moreover, there is now a clinical trial underway in which IGF-1 is being used for treatment of autism [103].

Therefore, normalizing IGF-1 levels might be one of the effects of IGF-1 therapy [104]. As stated above, functional defects in neuronal networks could be rescued by IGF [103]. IGF-1 reduces neuroinflammation by decreasing secretion of cytokines such as interleukin 6 (IL6) and alters microglial function, thereby reducing the defects in the synapses [23,100,105,106,107]. In autism there is aberrant control of synapse formation [23]. IGF-1 alleviates *N*-methyl-d-asparate (NMDA)-induced neurotoxicity via the IGF AKT (protein kinase B) -mammalian target of rapamycin (AKT-mTor pathway) [102]. This pathway is dysregulated in autism. IGF-1 may rescue function in RTT and ASD caused by changes of the SHANK3 gene [108,109,110], and reverse the reduction in excitatory synapse numbers [111].

## 5. What Are the Therapeutic Approaches?

During the last decade, there has been a dramatic increase in research aimed at treating neurodevelopmental disorders. These efforts have been the consequence of a better understanding of cellular mechanisms and the genetic basis of several of these disorders and the subsequent development of experimental models, primarily mouse models. The potential role of IGF-1 therapy would be in preventing neuronal injury, reducing neuronal degeneration and increasing myelination. In animals, IGF-1 has been shown to be effective in motor neuron disorder, neuropathy, cerebellar ataxia, ceroid lipofuscinosis; retinopathy of prematurity, and cognitive impairment in early autoimmune encephalopathy.

IGF-1 has been identified to have a crucial role in normal CNS development. Several clinical studies have suggested a potential role for IGF-1 in the treatment of disorders of CNS, including ASD. The focus of the recent studies has been to develop novel, targeted, and potentially disease modifying therapeutics in CNS diseases in children.

The major problems are the following: (1) Does enough IGF-1 cross the BBB? (2) Can the action of the drugs be lengthened? (3) Can we make the neurons active? (4) Are there serious side-effects?

Studies of RTT both in mouse models and humans show benefits of treatments with IGF-1, raising the possibility that this compound may have benefits broadly in ASD, despite the different molecular aetiology. Given the extensive safety data of IGF-1 therapy for IGF-1 deficiency-related short stature and a recent RTT study, IGF-1 is a good potential candidate for controlled trials in ASD [112,113]. 

### 5.1. What Is the Current Status of IGF-1 Treatment?

Recombinant human IGF-1 (mecasermin) was developed for the treatment of children with growth failure because of deficient IGF-1 liver production due to mutations or deletion of the hepatic growth hormone receptor [114,115]. The treatment has to be given until the end of puberty, when growth stops due to the ossification of the growth cartilage. Its commercial name is Increlex^®^ and it is given by subcutaneous injection. The main adverse effect of mecasermin is the risk of inducing hypoglycemia (it mimics most of the actions of insulin), and can have mitogen effects if given long-term [116]. Another problem is that it crosses with difficulty the BBB.

Despite these problems, IGF-1 has been used in patients with RTT (*n* = 6, aged 4–11 years), at doses of 0.05–0.1 mg/kg twice per day for six months, without significant adverse effects [117]. In this small pilot study, the parents of the patients observed that children with RTT showed improvement in cognition and social interaction. Two years later, the same group published the evolution of a RTT patient who was treated at the age of five years and eight months, with a first cycle of IGF-1 for four months and then two years later the same treatment was repeated [118]. Curiously, while scores from tests analyzing cognition and social abilities improved after each cycle, these achievements were lost during the two years without treatment. This indicates that the treatment of RTT with IGF-1 has to be maintained long-term. No adverse effects were reported, although the time during which the treatment took place was too short.

In the same year, it was reported that 12 girls with MECP2 mutations, nine of them with RTT, who were treated with IGF-1 experienced improvements in mood and anxiety. These findings were supported by reversal of right alpha band asymmetry on EEG, which is an established index of anxiety and depression. Moreover, these authors did not observe any adverse effects attributable to IGF-1, and also found that CSF IGF-1 levels had increased at the end of treatment period, which indicates that IGF-1 crosses the BBB [104].

In parallel with the aforementioned studies, a genetic subtype of autism was described [109]. This subtype of autism is known as Phelan–McDermic syndrome, which is produced by mutations or deletions in a gene, the SCHANK3 gene, located on the chromosome 22q, is responsible for the expression of a protein playing a critical role in synaptic function. Consequently, the loss of the function of that protein leads to a global developmental delay that cause cognitive disabilities, speech affections, hypotonia, and some dysmorphic features. The syndrome causes ASD-like behaviors, and is responsible for at least 0.5% of the cases of ASD. The authors carried out a placebo-controlled, double-blind, cross-over study in nine children (5–15 years old) who presented this syndrome, treating them with IGF-1 over three months, followed by three months of placebo after a four-week wash-out period. Before commencing the treatment and after finishing it, social impairments and restrictive behaviors, typical of the syndrome, were evaluated by the aberrant behavior checklist and the repetitive behaviour scale. Interestingly, after being treated with IGF-1 both scales showed significant improvements in the parameters evaluated, however these were not maintained after interrupting IGF-1 treatment [109].

A clinical trial with rhIGF-1 in children with Phelan–McDermic syndrome is currently underway ((ClinicalTrials. gov. Identifier: NCT0152901). IGF-1 (1–3) (NNZ-2566) Trofineitide, Neuren Pharmaceuticals).

Trofinetide is a synthetic analogue of a naturally occurring neurotrophic peptide derived from IGF-1. Endogenous peptidase enzymes cleave IGF-1, separating the terminal tripeptide IGF-1 (1–3). This peptide has been modified by methylation of glycine, allowing the drug to be administered orally or intravenously for a range of acute and chronic conditions. 

It crosses the BBB and easily enters into the brain. IGF-1 (1–3) does not bind directly to the IGF-1 receptors, but it acts through the IGF-1 receptor. IGF-1 (1–3) induces an increase in the production of IGF-1, which in turn activates the IGF-1 receptor [23]. This weak binding might mean that it is not a mitogen. The results also suggest that there is a direct interaction between both molecules.

IGF-1 (1–3) has considerably reduced binding to the IGFBPs, which results in an enhanced potency (about 10-fold in vivo) relative to IGF-1. This results in an increased prolongation of potential adverse events like hypoglycaemia [119]. However, NNZ-2566, Trofinetide is well-tolerated, and is currently being studied in clinical trials in at least two neurodevelopmental disorders, RTT (ClinicalTrials. gov. Identifier: NCT01703533) and Fragile X syndrome (FXS; ClinicalTrials. gov. Identifier: NCT01894958). It has been shown to rescue function of MECP2 in mouse model of RTT syndrome [101]. Since it has successfully passed Phase III clinical trials in adults, and is being studied in Phase II trials in children, it is expected that soon this new drug will be commercially available.

### 5.2. Longevity of the Therapy and Long-Term Adverse Effects

#### 5.2.1. How Long Should IGF-1 Therapy Be Used?

RTT syndrome is produced by mutations in the gene MECP2 encoding x-linked methyl CpCbinding protein. Therefore, it is likely that only a gene therapy would be able to revert this syndrome. However, treatments with IGF.1 have been shown to produce functional recoveries in MECP2 mutant mice [120], and, as described before, IGF-1 (1-3) administration induced partial reversal of RTT-like symptoms in humans [101]. However, the symptoms come back when the treatment is interrupted [118], which suggests that in this situation IGF-1 acts by epigenetic mechanisms. Since the disease begins early in postnatal life, it has been suggested that maintaining MECP2 function during the first stages of life might provide a kind of protection for preventing the apparition of the disease later in life. However, this has been proved to be not effective, as demonstrated by the McGraw group in an inducible model of RTT in which they improved the functionality of MECP2 during early postnatal life [121]. Therefore, the need exists for continuous IGF-1 administration to alleviate the symptoms of the disease, at least, until Trofinetide will be available. 

#### 5.2.2. Long-Term Side-effects

Although IGF-1 holds therapeutic promise, it may produce undesirable side effects and it must be used with caution. There seems to be an association between genetic variations in IGF-1 signalling pathways and cancer. High levels of circulating IGF-1 and certain genetic polymorphisms of IGF-1 and IGFBP3 increase the risk of several common cancers [122,123]. IGF-1 may also increase the risk of an abnormal metabolism and diabetes [124]. However, states of relative IGF-1 deficiency seem to be protective against cancer [125]. When plasma levels IGF-1 are low, mitogen effects seem not to occur. If hypoglycaemia occurs with recommended doses despite adequate food intake, the dose should be reduced. IGF-1 should be administered shortly before or after (±20 min) a meal or snack. During treatment with IGF-1 it is recommended to monitor its plasma concentration periodically, ensuring that its concentration does not markedly exceed 2 SD over the mean for the age of the patient, according the reference ranges [126].

### 5.3. Timing of Therapy

In the U.S. positive results have been reported by Neuren Pharma in adults with RTT treated with Trofinetide. Fifty-three patients aged 16 to 45 years completed the double-blind placebo-controlled trial. Of the six measures to ascertain change after treatment with the drug, three indicated a change for the better. Measures of impressions of motor behaviour involvement, and of the caregivers’ top three concerns, met the criteria of improvement, as reviewed by Pozzo-Miller et al. [127].

The drug should probably be given at an early age when there is a deficiency of IGF-1 in autism [51,52] and synaptic development is more active. The pathological process should be stopped at an early age before irreversible nerve growth damage occurs.

## 6. Conclusions

IGF-1 may be involved in the pathogenesis of some neurodevelopmental (RTT), and neurodegenerative diseases (PEHO, INCL, cerebellar degeneration and SSPE). IGF-1 may be neuroprotective in preterm infants, and after neonatal ischemic brain injury. In infantile spasms CSF IGF-1 studied at the time of presentation seems to be a biomarker of (1) treatment response and progression of epilepsy, and (2) of later cognitive outcome. Brain growth of autistic children correlates positively with serum and CSF IGF-1 concentrations. In ASD research molecular studies on synaptic dysfunction have proceeded to experimental therapeutics. The precise mechanism by which IGF-1 exerts its effects on the CNS remains an active area of study. Although definitive studies are needed, pilot data suggest that IGF-1 or its analogues are promising for the treatment of the neurodevelopmental disorders associated with ASD.
